# Clinical efficacy analysis of surgical treatment for spinal metastasis under the multidisciplinary team using the NOMS decision system combined with the revised Tokuhashi scoring system: a randomized controlled study

**DOI:** 10.1186/s13018-024-04668-1

**Published:** 2024-03-21

**Authors:** Xiao-Bing Xiang, Kai-Yin Gao, Wei-Wei Zhang, Cheng-Peng Li, Kai-Kai Feng, Guang-Ru Cao

**Affiliations:** The Second Affiliated Hospital of Zunyi Medical University, Orthopedics, Zunyi, Guizhou China

**Keywords:** Multidisciplinary team, NOMS scoring decision system, Revised Tokuhashi scoring system, Spinal metastasis, Surgical treatment, Quality of life

## Abstract

**Objective:**

Despite advancements in spinal metastasis surgery techniques and the rapid development of multidisciplinary treatment models, we aimed to explore the clinical efficacy of spinal metastasis surgery performed by a combined NOMS decision system-utilizing multidisciplinary team and Revised Tokuhashi scoring system, compared with the Revised Tokuhashi scoring system.

**Methods:**

Clinical data from 102 patients with spinal metastases who underwent surgery at three affiliated hospitals of Zunyi Medical University from December 2017 to June 2022 were analysed. The patients were randomly assigned to two groups: 52 patients in the treatment group involving the combined NOMS decision system-utilizing multidisciplinary team and Revised Tokuhashi scoring system (i.e., the combined group), and 50 patients in the treatment group involving the Revised Tokuhashi scoring system only (i.e., the revised TSS-only group). Moreover, there were no statistically significant differences in preoperative general data or indicators between the two groups. Intraoperative and postoperative complications, average hospital stay, mortality rate, and follow-up observation indicators, including the visual analogue scale (VAS) score for pain, Eastern Cooperative Oncology Group (ECOG) performance status, Karnofsky Performance Status (KPS) score, negative psychological assessment score (using the Self-Rating Anxiety Scale, [SAS]), and neurological function recovery score (Frankel functional classification) were compared between the two groups.

**Results:**

All 102 patients successfully completed surgery and were discharged. The follow-up period ranged from 12 to 24 months, with an average of (13.2 ± 2.4) months. The patients in the combined group experienced fewer complications such as surgical wound infections 3 patients(5.77%), intraoperative massive haemorrhage 2 patients(3.85%), cerebrospinal fluid leakage 2 patients(3.85%), deep vein thrombosis 4 patients(7.69%),and neurological damage 1 patient(1.92%), than patients in the revised TSS-only group (wound infections,11 patients(22%); intraoperative massive haemorrhage, 8 patients(16%);cerebrospinal fluid leakage,5 patients(10%);deep vein thrombosis,13 patients (26%); neurological damage,2 patients (4%). Significant differences were found between the two groups in terms of surgical wound infections, intraoperative massive haemorrhage, and deep vein thrombosis (P < 0.05). The average postoperative hospital stay in the combined group (7.94 ± 0.28 days) was significantly shorter than that in the revised TSS-only group (10.33 ± 0.30 days) (P < 0.05). Long-term follow-up (1 month, 3 months, 6 months, and 1 year postoperatively) revealed better clinical outcomes in the combined group than in the revised TSS-only group in terms of VAS scores, overall KPS%, neurological function status Frankel classification, ECOG performance status, and SAS scores.(P < 0.05).

**Conclusion:**

A multidisciplinary team using the NOMS combined with the Revised Tokuhashi scoring system for spinal metastasis surgery showed better clinical efficacy than the sole use of the Revised Tokuhashi scoring system. This personalized, precise, and rational treatment significantly improves patient quality of life, shortens hospital stay, reduces intraoperative and postoperative complications, and lowers mortality rates.

## Introduction

Bone metastasis is a common complication of cancer, caused by most types of malignancies, and frequently occurs in patients with breast cancer (65–75%), prostate cancer (65–90%), lung cancer (17–64%), or renal cancer (20–25%) [1]. The spine is the most common site for metastatic bone tumours, accounting for 60–70% of cases [2]. With in-depth research into primary tumours and spinal metastases, and improvements in medical oncology, surgical oncology, and radiotherapy, the survival time of cancer patients has increased, leading to a gradual increase in the incidence of spinal metastases. Spinal metastases often lead to complications such as spinal cord compression, spinal instability, pathological fractures, hypercalcaemia, intractable pain, severe disability or accelerated death, imposing a substantial burden on the healthcare system [3]. Treatment options for spinal metastases include radiotherapy, chemotherapy, surgical intervention, interventional therapy, ablation therapy, and targeted radionuclide therapy [4, 5]. However, the effectiveness of these treatments is often less than ideal due to various factors . As surgical techniques have developed, surgery has been found to play a crucial role in treating bone metastases from lung cancer. The primary goals of surgery are to alleviate and restore neurological function, reduce local pain from metastatic lesions, and control tumour progression. Surgery involves the complete removal of tumouor tissue and the restoration of normal physiological structure and stability of the spine, thereby effectively relieving tumouor compression and clinical symptoms [6]. The Revised Tokuhashi scoring system is widely regarded as the most extensive and effective system for evaluating spinal metastases [7]. In recent years, with the rapid development of multidisciplinary teams (MDTs) in clinical settings, important achievements have been made in the treatment of spinal metastases [8]. The accuracy of the traditional modified Tokuhashi scoring system has been gradually reduced, and it fails to provide more suitable treatment plans. Under the multidisciplinary team (MDT) approach, a more comprehensive NOMS scoring decision system has been developed for the treatment of spinal metastasis; this system evaluates and determines treatment options for each patient based on neurological function, oncological characteristics, spinal stability, and overall condition, addressing the limitations of the modified Tokuhashi scoring system and its sole prediction of patient survival [9]. However, the NOMS does not consider patient prognosis, and there are still limitations in guiding treatment selection. Therefore, a combination of multiple scoring decision systems by a multidisciplinary team is used to personalize and standardize treatment plans based on various indicators, such as expected survival, physical condition, neurological function, oncological characteristics, and spinal stability, aiming to maximize patients’ quality of life and extend their lives, which is the goal of clinical treatment [10]. To date, there have been no reports on clinical comparative studies between the NOMS combined with the Revised Tokuhashi scoring system and the Revised Tokuhashi scoring system alone in the surgical treatment of spinal metastases under a multidisciplinary team approach. Hence, in this study, we analyse and report on the clinical efficacy of these two approaches in 102 patients with spinal metastases treated surgically at three affiliated hospitals of Zunyi Medical University from December 2017 to June 2022.

## Materials and methods

### Study subjects

This study adhered to the CONSORT guidelines. This randomized controlled study involved 102 patients with spinal metastases treated at three affiliated hospitals of Zunyi Medical University from December 2017 to June 2022. Patients were randomly assigned to two groups using a random number table : 52 patients in in the treatment group involving the combined NOMS decision system-utilizing multidisciplinary team and Revised Tokuhashi scoring system (i.e., the combined group)and 50 patients in the treatment group involving the Revised Tokuhashi scoring system only (i.e., the revised TSS-only group). The study was approved by the Ethics Committee of the affiliated hospitals of Zunyi Medical University. All patients signed an informed consent form and voluntarily participated in the study.

Inclusion/exclusion criteria for patients were as follows: (1) ≥ 18 years of age; (2) definite diagnosis of spinal metastases and willingness to undergo selected surgical treatment; (3) clear primary tumour, including lung cancer, breast cancer, prostate cancer, or kidney cancer; (4) follow-up duration after surgery ≥ 1 year with complete follow-up data; (5) expected survival period ≥ 6 months; (6) good general condition to tolerate surgery; (7) treated by experienced spine surgeons; and (8) having the surgical indications: (a) presence of spinal instability—Spinal Instability Neoplastic Score (SINS) ≥ 7 points, (b) presence of progressive neurological compression symptoms—epidural spinal cord compression (ESCC) grade ≥ 2, and (c) presence of refractory and intractable pain. The exclusion criteria for patients were as follows: (1) primary spinal tumours; (2) complications such as vertebral fractures and severe intervertebral disc herniation caused by factors other than metastatic tumours which may affect postoperative evaluation; (3) lack of complete medical history and/or loss to follow-up after treatment; (4) life expectancy < 6 months and responsive to narcotic analgesics or markedly responsive to radiotherapy; poor general condition (Karnofsky Performance Status 3 or poorer); or reduced will to live.

### Preoperative clinical data in the two groups

All patients underwent comprehensive preoperative examinations. The collected data included name, sex, age, type of primary tumouor, metastasis location, SINS (0–18 points, with 0–6 indicating spinal stability, 7–12 indicating potential instability, and 13–18 indicating instability; a score ≥ 7 points indicated surgical treatment), ESCC grade ( grade 0: tumouor confined to bone; grade 1: tumouor extends to extradural space without deforming the spinal cord; grade 2: spinal cord compression but cerebrospinal fluid visible; grade 3: spinal cord compression with no visible cerebrospinal fluid), preoperative VAS score (0–10 points, with higher scores indicating more severe pain), Eastern Cooperative Oncology Group (ECOG) performance status (0–5 grades, with higher grades indicating worse physical condition; level 0, normal activity capacity, no difference compared to preonset activity level; level 1, able to freely move and engage in light physical activities, including general household chores or office work, but unable to perform heavy physical activities; level 2, ability to freely move and perform activities of daily living but with lost work capacity; can be out of bed and active for at least half of the day; level 3, partially able to perform activities of daily living, spending more than half of the day in bed or wheelchair; level 4, bedridden, unable to perform activities of daily living independently; level 5, death), overall condition Karnofsky Performance Scale (KPS) score (0-100%, divided into ten grades, with higher scores indicating better physical condition), and negative psychological assessment score (using the self-rating anxiety scale (SAS), anxiety levels can be classified into four categories: normal, mild, moderate, and severe anxiety; normal, SAS score < 50; mild, SAS score between 50 and 59; moderate, SAS score between 60 and 69; severe, SAS score ≥ 70), neurological function status,Frankel classification, divided into A, B, C, D and E; 5 grades, with A, complete paralysis; B, incomplete loss of sensory function, no motor function; C, incomplete loss of sensory function, nonfunctional movement; D, incomplete loss of sensory function, functional movement; and E, normal sensory and motor function).

### Study methods

#### Multidisciplinary team using NOMS and revised Tokuhashi scoring system group

**Fig. 1:**
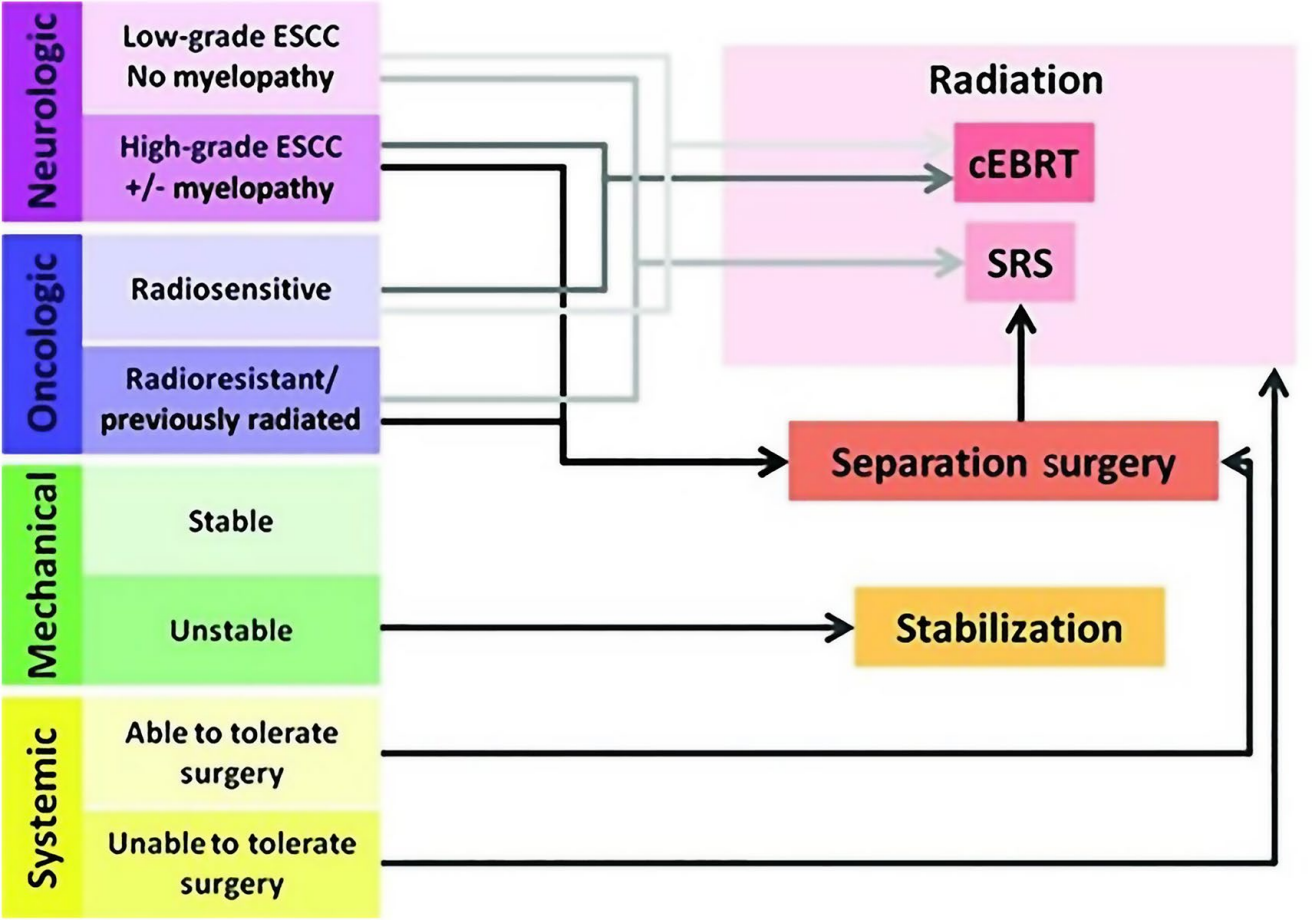
Schematic depiction of the neurologic, oncologic, mechanical, and systemic (NOMS) decision framework [9]

**(1) Establish the multidisciplinary team**: Led by orthopaedic surgeons, the team includes general surgery, oncology, radiology, pathology, pain management, nuclear medicine, endocrinology, rehabilitation, psychosomatic medicine, and anaesthesiology staff. Personnel from each specialty play a specific role, with orthopaedic surgeons overseeing and implementing the entire treatment plan, including surgical procedures. **(2) Preoperative assessment and adjustment by the MDT**: Upon admission, the team evaluated and adjusted the patient’s preoperative condition (correcting hypoproteinaemia, adjusting blood pressure, controlling blood sugar, regulating blood clotting function, and providing psychological support to enhance self-confidence) based on various indices such as primary tumouor, preoperative examination results, SINS, ESCC grade, VAS score, ECOG physical condition, KPS score, SAS score, and Frankel classification. The team collaboratively formulated the preoperative treatment plan and prepared the patient for surgery. **(3) Surgical plan formulated and executed by the MDT**: Based on the aforementioned indices, the MDT thoroughly discussed and strictly followed the NOMS decision system and the Revised Tokuhashi scoring system to assess each patient and develop a comprehensive surgical plan, choosing between excisional surgery and postoperative traditional external beam radiotherapy (cEBRT) or stereotactic radiosurgery (SRS), depending on the radiation sensitivity, ESCC grade, SINS, and the Revised Tokuhashi score. (a) For patients with ESCC grade ≥ 2, SINS ≥ 7 points, Revised Tokuhashi score ≥ 12 points, and a general condition tolerant of surgery, if radiotherapy-sensitive, en-bloc resection surgery and postoperative conventional external beam radiation therapy (cEBRT), if radiotherapy-insensitive, en-bloc resection surgery and postoperative stereotactic radiosurgery (SRS) are selected; (b) For patients with ESCC grade ≥ 2, SINS ≥ 7, Revised Tokuhashi score of 9–11, and systemic conditions who are surgery-tolerant, palliative surgery and postoperative traditional external beam radiation therapy (cEBRT) if radiotherapy-sensitive, and palliative surgery and postoperative stereotactic radiosurgery (SRS) if radiotherapy-insensitive; (c) For patients with ESCC grade < 2, SINS ≥ 7 points, Revised Tokuhashi score ≥ 12 points, and systemic conditions that tolerate surgery, if radiotherapy-sensitive, en-bloc resection surgery and postoperative conventional external beam radiation therapy (cEBRT) are selected, if radiotherapy-insensitive, en-bloc resection surgery and postoperative stereotactic radiosurgery (SRS) are selected; (d) For patients with ESCC grade < 2, SINS score of ≥ 7, Revised Tokuhashi score of 9–11, and systemic surgery-tolerant, if radiotherapy-sensitive, palliative surgery and postoperative conventional external beam radiation therapy (cEBRT) are selected, if radiotherapy-insensitive, palliative surgery and postoperative stereotactic radiosurgery (SRS) are selected.) (4) Multidisciplinary collaboration in surgery execution. (5) Postoperative care by the MDT: Infection prevention, psychological therapy, pain management, dietary and nutritional therapy, rehabilitation and functional training, and further treatment of the primary tumouor (radiotherapy, chemotherapy, and targeted therapy). (6) Discharge planning by the MDT: includes detailed plans for radiotherapy, chemotherapy, psychological therapy, rehabilitation training, and regular follow-up scheduling. (Fig. 1; Tables 1 and 2).

**Table 1:** Revised Tokuhashi prognostic score

	Score 0	Score 1	Score 2	Score3	Score 4	Score 5
Karnofsky’s performance (%)	10–40	50–70	80–100			
Extraspinal bone metastases	3 or more	1–2	0			
Vertebral metastases	3 or more	2	1			
Visceral metastases	Unremovable	Removable	None			
Primary site (e.g.)	Lung	Liver	Other	Kidney	Rectum	Breast
Palsy	Frankel A, B	Frankel C, D	Frankel E			

The scores for the six individual criteria mentioned above are summed to provide a total score, with a maximum of 15. Tokuhashi and colleagues suggest that patients with a favorable prognosis (scoring 12–15) undergo resection surgery, with an expected survival period of ≥ 12 months. Most patients with a moderate prognosis (scoring 9–11) undergo palliative surgery, with an expected survival period of ≥ 6 months. Those with a poor prognosis (scoring 0–8) are recommended conservative treatments, with an expected survival period of < 6 months

**Table 2:** Treatment selection table for NOMS combined with revised Tokuhashi scoring system

Neurologic(N)	Oncologic(O)	Mechanical(M)	Sytemic(S)	Revised Tokuhashi		Decision
ESCC grade ≥ 2	Radiosensitive	SINS ≥ 7	Able to tolerate surgery	≥ 12	en-bloc resection and cEBRT	
ESCC grade ≥ 2	Radioresistant	SINS ≥ 7	Able to tolerate surgery	≥ 12	en-bloc resection and SRS	
ESCC grade ≥ 2	Radiosensitive	SINS ≥ 7	Able to tolerate surgery	9–11	Palliative surgery and cEBRT	
ESCC grade ≥ 2	Radioresistant	SINS ≥ 7	Able to tolerate surgery	9–11	Palliative surgery and SRS	
ESCC grade<2	Radiosensitive	SINS ≥ 7	Able to tolerate surgery	≥ 12	en-bloc resection and cEBRT	
ESCC grade<2	Radioresistant	SINS ≥ 7	Able to tolerate surgery	≥ 12	en-bloc resection and SRS	
ESCC grade<2	Radiosensitive	SINS ≥ 7	Able to tolerate surgery	9–11	Palliative surgery and cEBRT	
ESCC grade<2	Radioresistant	SINS ≥ 7	Able to tolerate surgery	9–11	Palliative surgery and SRS	

#### Solely treated according to the revised Tokuhashi scoring system group

##### (1) Orthopaedic surgeons independently adjusted the preoperative condition

Based on various indices such as the primary tumouor, preoperative examination results, SINS score, ESCC grade, VAS score, ECOG physical condition, KPS score, SAS score, and Frankel classification, orthopaedic surgeons developed the preoperative treatment plan and adjust the patient’s condition, with oncological consultations for primary and metastatic lesions and anaesthesia evaluation one day before surgery. There was no involvement of pain management, psychosomatic medicine, radiology, or rehabilitation departments. (2) Orthopaedic surgeons independently formulated and executed the surgical plan. Strictly following the Revised Tokuhashi scoring system for patient assessment, surgeons chose between excisional and palliative surgery based on the Revised Tokuhashi score. (3) Postoperative management by orthopaedic surgeons included fluid replacement, anti-infection measures, empirical pain control, and rehabilitation exercises, with consultations from relevant departments as necessary. (4) Poststabilization, regular radiotherapy and chemotherapy plans were developed for tumouor treatment.

#### Observed indices

Patients are regularly followed up in the outpatient clinic at 1, 3, 6, and 12 months after surgery. The two groups of patients with the same primary type of tumours were given the same type of chemoradiotherapy.

##### Intraoperative, postoperative, and follow-up observation indices included

intraoperative or postoperative complications, average postoperative hospital stay (days), and mortality rate.

**Long-term postoperative follow-up assessments at 1, 3, 6, and 12 months included the following**:(1)VAS score: The patient’s postoperative pain status was evaluated (0–10 points, with higher scores indicating more severe pain). (2) ECOG Performance Status: The recovery of the patient’s physical condition after tumouor surgery was assessed (0–5 grades, with higher grades indicating worse physical condition). (3) Karnofsky Performance Scale (KPS%) score: This score was used to assess the patient’s e ability to perform daily activities postoperatively and quality of life (0-100%, divided into ten grades, with higher scores indicating better physical condition). (4) Self-rating anxiety scale (SAS) score: The SAS was used to evaluate the patient’s postoperative psychological state (divided into normal, mild, moderate, and severe). (5) Neurological Function Status (Frankel Classification): Graded as A, B, C, D and E (A: Complete paralysis; B: Incomplete loss of sensory function, no motor function; C: Incomplete loss of sensory function, nonfunctional movement; D: Incomplete loss of sensory function, functional movement; E: Normal sensory and motor function).

#### Statistical methods

**Fig. 2:**
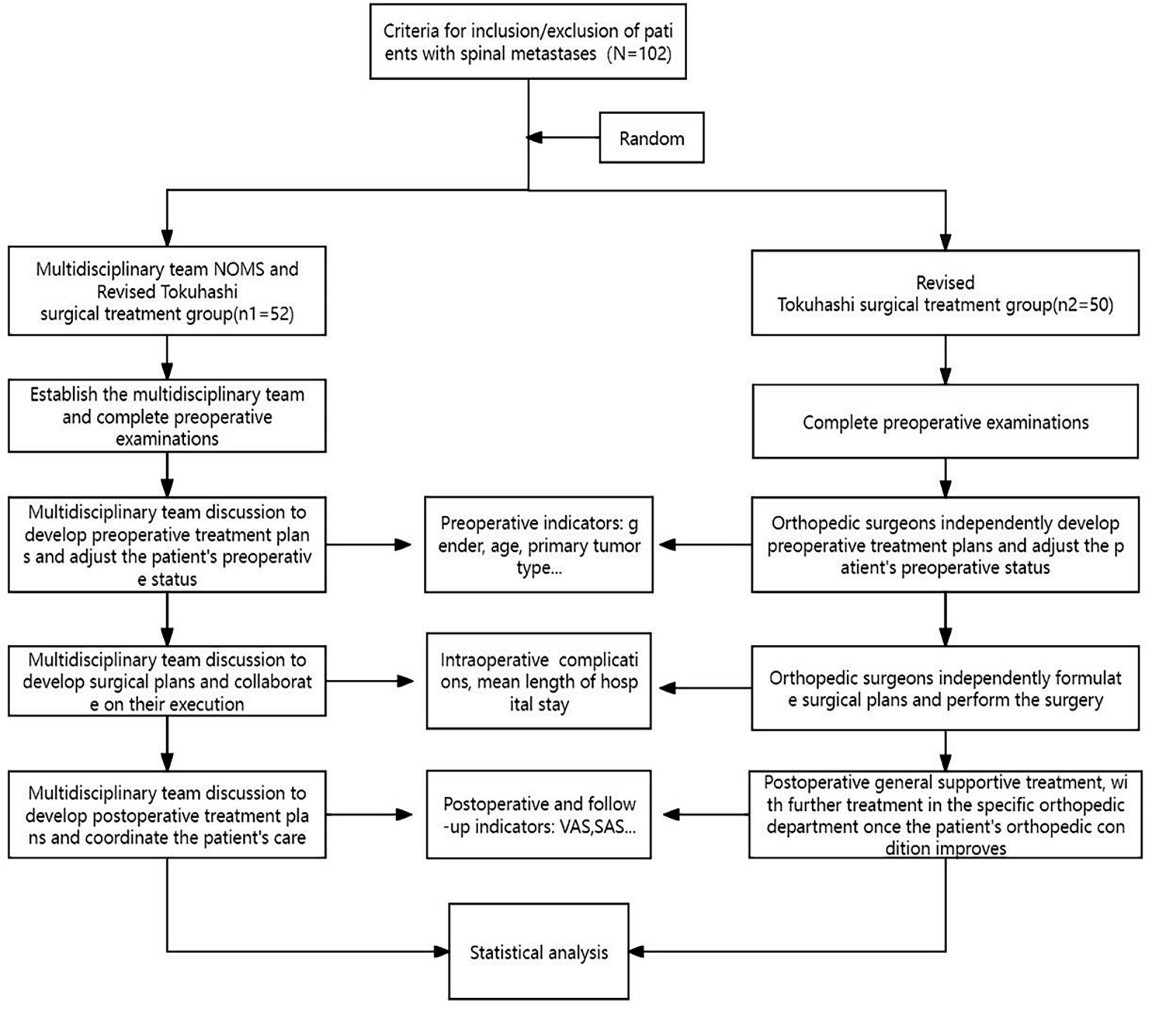
Flow chart

We used SPSS 25.0 (IBM Corp, Armonk, NY, USA) software for statistical analysis. Continuous data were presented as mean ± standard deviation (x ®±s). Independent sample t-test or Mann-Whitney U-test was performed for quantitative data such as age, VAS score, KPS score, SINS score. Chi-square test, Fisher’s exact test, or rank-sum test were used for categorical data such as ECOG performance status, Frankel grade, SAS score categories, gender. P < 0.05 was considered to indicate statistical significance (See Fig. 2).

## Results

### Preoperative clinical data of the two groups: no statistically significant differences

A multidisciplinary team from the NOMS jointly improved the Tokuhashi scoring system for surgical treatment in a group of 52 patients, including 27 males (51.92%) and 25 females (48.08%). The distribution of cancer types in this group was as follows: 24 had lung cancer (46.15%), 15 had breast cancer (28.85%), 5 had kidney cancer (9.62%), and 8 had prostate cancer (15.38%). The average age of the patients was 60.04 ± 4.61 years. The sites of metastasis invasion in the spine were as follows: 8 in the cervical spine (15.38%), 2 in the cervicothoracic spine (3.85%), 22 in the thoracic spine (42.31%), 3 in the thoracolumbar spine (5.77%), and 17 in the lumbar spine (32.69%). The Spinal Instability Neoplastic Score (SINS) was recorded as 13.10 ± 1.88. Prior to surgery, the patients’ neurological function was assessed using the Frankel classification: A, 0 patients; B. 0 patients; C, 10 patients (19.23%), D, 27 patients (51.92%); and E, 15 patients (28.85%). The preoperative visual analogue scale (VAS) score was 6.13 ± 0.79. The patients’ physical status prior to surgery was evaluated using the Eastern Cooperative Oncology Group (ECOG) performance status classification: Grade 0, 0 cases; Grade 1, 0 cases; Grade 2, 28 cases (53.85%); Grade 3, 19 cases (36.54%); Grade 4, 5 cases (9.61%); Grade 5, 0 cases. The preoperative Karnofsky Performance Scale (KPS) score was 48.88 ± 6.44. The patients’ self-assessment of anxiety levels prior to surgery, measured using the Self-Rating Anxiety Scale (SAS), showed the following distribution: normal, 0 patients; mild, 19 patients (36.54%); moderate, 27 patients (51.92%); severe, 6 patients (11.54%). In a separate group in which the Tokuhashi scoring system was modified alone, there were 50 patients, 23 males (46%) and 27 females (54%). The distribution of cancer types in this group was as follows: 21 had lung cancer (42%), 13 had breast cancer (26%), 9 had kidney cancer (18%), and 7 had prostate cancer (14%). The average age of the patients was 60.92 ± 5.28 years. The sites of metastastic invasion in the spine were as follows: 12 in the cervical spine (24%), 1 in the cervicothoracic spine (2%), 21 in the thoracic spine (42%), 2 in the thoracolumbar spine (4%), and 14 in the lumbar spine (28%). The Spinal Instability Neoplastic Score (SINS) was recorded as 12.74 ± 1.956. Prior to surgery, the patients’ neurological function was assessed using the Frankel classification: A, 0 patients; B, 0 patients; C, 7 patients (14%); D, 25 patients (50%); and E, 18 patients (36%). The preoperative visual analogue scale (VAS) score was 6.15 ± 0.81. The patients’ physical statuses prior to surgery were evaluated using the Eastern Cooperative Oncology Group (ECOG) performance status classification: Grade 0, 0 cases; Grade 1, 0 cases; Grade 2, 29 cases (58%); Grade 3, 18 cases (36%); Grade 4, 3 cases (6%); Grade 5, 0 cases. The preoperative Karnofsky Performance Scale (KPS) score was 50.00 ± 7.25. The patients’ self-assessment of anxiety levels prior to surgery, measured using the Self-Rating Anxiety Scale (SAS), showed the following distribution: normal, 0 cases; mild, 21 cases (42%); moderate, 25 cases (50%); severe, 4 cases (8%). Preoperative general clinical data comparison between the two groups, including sex, age, type of primary tumour, spinal metastasis location, SINS score, ESCC grade, preoperative VAS score, preoperative Karnofsky Performance Scale (KPS) score, preoperative Eastern Cooperative Oncology Group (ECOG) performance status, preoperative SAS score, and neurological function status according to the Frankel classification, revealed no significant differences (P > 0.05), indicating comparability between the two groups (Table 3).

**Table 3:** Comparison of preoperative general data between the two groups

Category	NOMS + Revised Tokuhashi Group	Revised Tokuhashi	P
Number of Cases(n)	52	50	
Gender (n,%)			0.55
Male	27(51.92)	23(46)	
Female	25(48.08)	27(23)	
Age (years)	60.04 ± 4.61	60.92 ± 5.82	0.17
Type of Primary Tumor (n,%)			0.68
lung cancer	24(46.15)	21(42)	
Breast cancer	15(28.85)	13(26)	
Renal cancer	5(9.62)	9(18)	
Prostate cancer	8(15.38)	7(14)	
Metastasis Site			0.81
Cervical	8(15.38)	12(24)	
Cervicothoracic	2(3.85)	1(2)	
Thoracic	22(42.31)	21(42)	
Thoracolumbar	3(5.77)	2(4)	
Lumbar	17(32.69)	14(28)	
SINS	13.10 ± 1.88	12.74 ± 1.956	0.35
Frankel Classification			0.64
A and B grades	0(0)	0(0)	
C grade	10(19.23)	7(14)	
D grade	27(51.92)	25(50)	
E grade	15(28.85)	18(36)	
Preoperative VAS Score	6.13 ± 0.79	6.15 ± 0.81	0.85
Preoperative KPS Score (%)	48.88 ± 6.44	50.00 ± 7.25	0.27
Preoperative ECOG Physical Condition Score			0.45
0 and 1 levels	0(0)	0(0)	
2 level	28(53.85)	29(58)	
3 level	19(36.54)	18(36)	
4 level	5(9.61)	3(6)	
5 level	0(0)	0(0)	
Preoperative SAS Score			0.76
Normal	0 (0)	0(0)	
Mild	19(36.54)	21(42)	
Moderate	27(51.92)	25(50)	
Severe	6(11.54)	4(8)	

Data are presented as n (%) or mean ± standard deviation

There was no difference in all general clinical data between the two groups preoperatively, which was not statistically significant (P > 0.05)

### Treatment methods

According to the multidisciplinary team NOMS combined with the modified Tokuhashi score, 5 patients (9.62%) underwent total vertebrectomy, with 3 of them receiving stereotactic radiotherapy and 2 receiving conventional external beam radiotherapy; 23 patients (44.23%) underwent tumour reduction surgery, with 10 of them receiving stereotactic radiotherapy and 13 receiving conventional external beam radiotherapy; and 24 patients (46.15%) underwent palliative decompression and fixation surgery, with 11 of them receiving stereotactic radiotherapy and 13 receiving conventional external beam radiotherapy. In the simple modified Tokuhashi score decision system surgical treatment group of 50 patients, 8 patients (16%) underwent total vertebrectomy, 20 patients (40%) underwent tumour reduction surgery, and 22 patients (40%) underwent palliative decompression and fixation surgery.

### Fewer intraoperative and postoperative complications were reported by the multidisciplinary team in the NOMS and revised Tokuhashi scoring system group

A comparison of intraoperative and postoperative complications between the two groups revealed that compared with patients treated solely with the Revised Tokuhashi scoring system, patients treated with the multidisciplinary team using the NOMS and Revised Tokuhashi scoring systems had lower rates of surgical site infection (5.77% vs. 22%,p = 0.017), massive intraoperative bleeding (3.85% vs. 16%,p = 0.039), cerebrospinal fluid leakage (3.85% vs. 10%,p = 0.219), deep vein thrombosis (7.69% vs. 26%,p = 0.013), and neurological damage (1.92% vs. 4%,p = 0.535). Surgical site infection, massive intraoperative bleeding and deep vein thrombosis were significantly lower in combined group(P < 0.05). Additionally, the average postoperative hospital stay was significantly shorter in the combined group (7.94 ± 0.28 days) than in the Revised Tokuhashi group (10.33 ± 0.30 days) (P = 0.037) (Table 4).

**Table 4:** Comparison of intraoperative and postoperative complications, and postoperative hospital stay between the two groups

Category	NOMS + Revised Tokuhashi Group	Revised Tokuhashi	P
Surgical Site Infection n (%)	3(5.77%)	11(22%)	0.017<0.05
Intraoperative Massive Bleeding n (%)	2 (3.85%)	8 (16%)	0.039<0.05
Cerebrospinal Fluid Leakage n (%)	2(3.85%)	5(10%)	0.219
Deep Vein Thrombosis n (%)	4(7.69%)	13(26%)	0.013<0.05
Neurological Damage n (%)	1(1.92%)	2(4%)	0.535
Postoperative Hospital Stay (days)	7.94 ± 0.28	10.33 ± 0.30	0.037<0.05

### The patients in the combined group achieved better clinical efficacy in the long-term follow-up

**Fig. 3:**
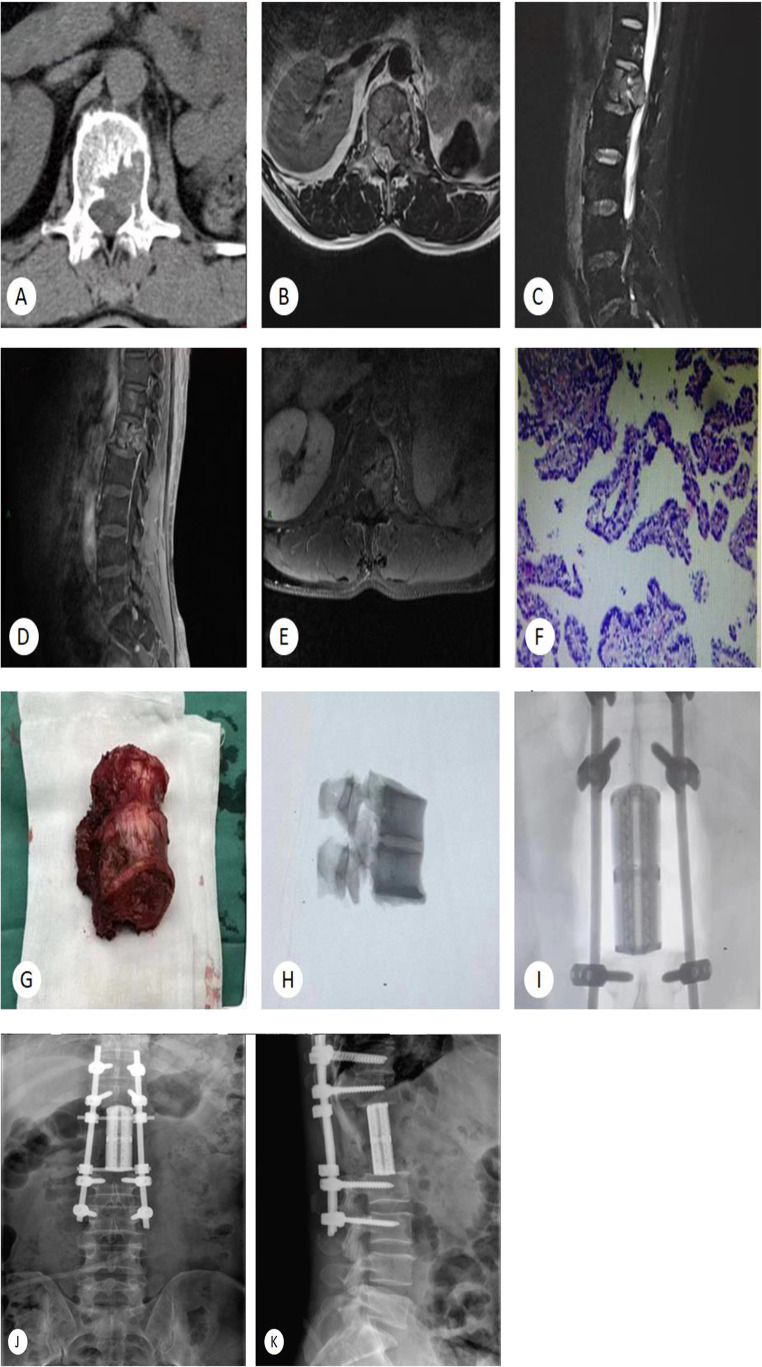
Typical case study

In the long-term follow-up, compared with those in the Revised Tokuhashi group, patients in the combined group had significantly better clinical outcomes lower VAS scores, higher Karnofsky Performance Scale (KPS%) scores, better neurological function (Frankel classification), better Eastern Cooperative Oncology Group (ECOG), and lower SAS scores (P < 0.05) (Table 5, See Fig. 3).

**Table 5:** Comparison of long-term postoperative follow-up indicators and mortality rate between the two groups

Category	NOMS + Revised Tokuhashi Group	Revised Tokuhashi	P
VAS Score			
post-surgery 1 month	1.72 ± 0.63	2.05 ± 0.60	0.027
post-surgery 3 month	0.84 ± 0.81	1.23 ± 0.87	0.035
post-surgery 6 month	0.22 ± 0.42	0.51 ± 0.56	0.018
post-surgery 12 month	0.68 ± 0.72	1.22 ± 0.94	0.024
KPS Score (%)			
post-surgery 1 month	64.38 ± 7.59	59.49 ± 7.59	0.013
post-surgery 3 month	78.44 ± 9.87	73.33 ± 10.60	0.021
post-surgery 6 month	86.88 ± 22.50	80.25 ± 23.00	0.032
post-surgery 12 month	94.64 ± 6.37	91.56 ± 5.74	0.040
Frankel Classification(n,%)			
post-surgery 1 month A and B grades C grade D grade E grade	0(0) 2(3.85) 23(44.23) 27(51.92)	0(0) 4(8) 23(46) 23(46)	0.441 > 0.05
post-surgery 3 month A and B grades C grade D grade E grade	0(0) 0(0) 14(26.92) 38(73.08)	0(0) 2(4) 23(46) 25(50)	0.033
post-surgery 6 month A and B grades C grade D grade E grade	0(0) 0(0) 8(15.38) 44(84.62)	0(0) 2(4) 18(36) 30(60)	0.043
post-surgery 12 month A,B and C grades D grade E grade	2 deaths(3.85) 0(0) 5(9.61) 45(86.54)	8 deaths(16) 0(0) 12(24) 30(60)	0.022
ECOG Physical Condition Score(n,%)			
post-surgery 1 month 0 level 1 level 2 level 3 level 4 level 5 level	0(0) 11(21.15) 26(50) 13(25) 2(3.85) 0(0)	0(0) 3(6) 25(50) 22(44) 0(0) 0(0)	0.031
post-surgery 3 month 0 level 1 level 2 level 3 level 4 and 5 levels	6(11.55) 17(32.69) 21(40.38) 8(15.38) 0(0)	1(2) 10(20) 23(46) 16(32) 0(0)	0.044
post-surgery 6 month 0 level 1 level 2 level 3 level 4 and 5 levels	11(21.15) 20(38.46) 19(36.54) 2(3.85) 0(0)	4(8) 14(28) 22(44) 10(20) 0(0)	0.037
post-surgery 12 month 0 level 1 level 2 level 3,4 and 5 levels	2 deaths(3.85) 19(36.54) 26(50) 5(9.61) 0(0)	8 deaths(16) 7(14) 22(44) 13(26) 0(0)	0.028
SAS Score(n,%)			
post-surgery 1 month Normal Mild Moderate Severe	20(38.46) 22(42.31) 10(19.23) 0(0)	13(26) 15(30) 20(40) 2(4)	0.035
post-surgery 3 month Normal Mild Moderate Severe	36(69.23) 11(21.15) 5(9.62) 0(0)	20(40) 21(42) 9(18) 0(0)	0.042
post-surgery 6 month Normal Mild Moderate Severe	40(76.92) 12(23.08) 0(0) 0(0)	26(52) 19(38) 5(10) 0(0)	0.030
post-surgery 12 month Normal Mild Moderate Severe	2 deaths(3.85) 44(84.61) 6(11.54) 0(0) 0(0)	8 deaths(16) 30(60) 10(20) 2(4) 0(0)	0.047
1-Year Postoperative Mortality n (%)	2 (3.85%)	8 (16%)	0.039

Data are presented as n (%) or mean ± standard deviation. Only post-surgery 1 month Frankel Classification P > 0.05, There were statistically significant comparisons of other Long-Term Postoperative Follow-Up Indicators and Mortality Rate Between the Two Groups P < 0.05

A 52-year-old male patient with metastasis of kidney cancer led to bone destruction of the thoracic 12th and 1st lumbar vertebrae. After evaluation by the multidisciplinary team using the NOMS combined with Revised Tokuhashi scoring system, the patient was scheduled for surgery under general anesthesia. The surgery consisted of resection of the tumouor lesions of the 12th thoracic vertebra and 1st lumbar vertebrae, decompression of the spinal canal, removal of the intervertebral discs, reconstruction of the intervertebral space with 3D printed artificial vertebral bodies, and fixation with pedicle screws and rod systems. After regular postoperative chemoradiotherapy, the patient recovered well. A: CT of thoracic spine showed bone destruction of the T12 vertebral bodies. B-E: MRI of thoracolumbar spine showed a primary tumour or metastatic lesion in the L1 vertebral body, abnormal signals in the T12 vertebral body and the left kidney is absent. F: pathological puncture revealed glandular-like glandular-like, papillary arranged, cuboidal or columnar epithelial cells, indicating tumour lesions. G-I: intra-operative photos. J-K: post-operative radiography.

## Discussion

This study, conducted by a multidisciplinary team, for the first time, combines a classification-based prognostic model with an improved Tokuhashi scoring system and a principle-based NOMS scoring decision system to prospectively select surgical methods and postoperative chemotherapy regimens for 52 patients with spinal metastases. The results were compared with those of a random group of 50 patients for whom only the improved Tokuhashi scoring system was used to determine treatment options. The findings indicate that the multidisciplinary team, in conjunction with the NOMS combined with the improved Tokuhashi scoring decision system, can provide better surgical methods and postoperative radiotherapy plans for spinal metastases. This approach also leads to lower intraoperative and postoperative complications as well as improvements in long-term pain symptoms and neurological function. Additionally, it effectively improves patient anxiety levels, resulting in positive clinical outcomes.

### Limitations of the revised Tokuhashi scoring system and NOMS decision system in treating spinal metastasis

There is still controversy surrounding the selection of treatment options and surgical indications for spinal metastasis. Traditionally, it is believed that patients with spinal instability or neurological or spinal cord compression who can tolerate surgery and have a longer expected survival time (≥ 6 months) often require surgical treatment [11]. In 2005, the modified Tokuhashi scoring system was first proposed to assess the choice of treatment options and achieved good therapeutic effects [12]. However, with advancements in tumour research, surgical techniques, radiation therapy, chemotherapy, and the application of multidisciplinary teams, the clinical efficacy of the modified Tokuhashi scoring system has gradually declined over time [13]. With respect to the surgical treatment group analysed using only the modified Tokuhashi scoring system, we found that the treatment plans of all 50 patients were independently determined by orthopaedic surgeons without sufficient collaboration from related departments. Multiple aspects, such as neurologic function, tumour characteristics, spinal stability, and overall condition, were not fully evaluated, resulting in the inability to establish a more effective treatment system. Furthermore, in the surgical treatment group involving only the modified Tokuhashi scoring system, there were greater incidences of complications, including major bleeding in 8 patients (16%), postoperative wound infections in 11 patients (22%), deep vein thrombosis in 13 patients (26%), cerebrospinal fluid leakage in 5 patients (10%), and neurological deficits in 2 patients (4%) [14]. These findings are consistent with those of a retrospective analysis conducted by Lee et al., who reported common complications in 200 patients with spinal metastasis; however, Lee et al. considered wound infection (30%) to be the most common complication. This differs from our study, where deep vein thrombosis (26%) was considered the most common complication. In terms of mortality, the one-year mortality rate in the surgical treatment group involving only the modified Tokuhashi scoring system was 16%, which was higher than the 3.85% in the surgical treatment group involving the multidisciplinary team-based NOMS (neurologic, oncologic, mechanical, and systemic) combined with the modified Tokuhashi scoring decision system. However, the one-year mortality rate in the surgical treatment group involving only the modified Tokuhashi scoring system was lower than the 22.2% reported by Yao et al. for 54 patients with breast cancer spinal metastasis [15]. This may be attributed to differences in the study subjects and the relatively small sample sizes of both studies, which included approximately 50 patients. With the development of tumour treatment modalities and the application of molecular targeted drugs, the accuracy of the modified Tokuhashi scoring system has also declined over the years. In a nationwide study conducted in France, the accuracy rates for survival prediction using the modified Tokuhashi and Tomita scoring systems were 42.8% and 25.6%, respectively [11]. With the application of multidisciplinary teams, the NOMS decision treatment model was first introduced in 2006. Patients were evaluated for neurological function (N), oncological status (O), spinal stability (M), and overall condition (S). Compared to the modified Tokuhashi scoring system, the NOMS decision system can better adapt to evolving treatment modalities and provide timely guidance for selecting appropriate treatment methods. However, the NOMS decision system does not consider patient prognosis and cannot predict patient survival [9].

### Advantages of a multidisciplinary team using NOMS and revised Tokuhashi scoring system in treating spinal metastasis

The multidisciplinary team (MDT) model, which originated in the 1990s, is based on the core concept of patient-centred care. It focuses on specific diseases and relies on multidisciplinary teams to develop standardized, individualized, and continuous comprehensive treatment plans [16]. After nearly 30 years of development, the MDT model has not only achieved excellent results in the treatment of tumours but also plays a vital role in the treatment of other diseases, including the involvement of other clinical departments. Therefore, the MDT model has become one of the most commonly used methods in clinical consultations and diagnostic processes in today’s medical field and represents an inevitable trend in the development of modern medicine [17]. The MDT model can effectively overcome barriers between disciplines and is a suitable approach for interdisciplinary collaboration in clinical settings. Patients’ conditions were comprehensively assessed to facilitate the systematic determination of diagnostic and treatment plans [18].

We conducted a randomized controlled study involving two groups: the surgical treatment group, for which a combined improved Tokuhashi scoring decision system was used by a multidisciplinary team (MDT), and the surgical treatment group, for which a simple improved Tokuhashi scoring system was used. The results demonstrated that the MDT approach, which incorporates both decision systems, formed a more effective and comprehensive treatment system. Among the 52 patients with spinal metastases, 2 (3.85%) had major intraoperative bleeding, 3 (5.77%) had postoperative wound infection, and 4 (7.69%) had deep vein thrombosis. These complications occurred at significantly lower rates than in the group treated with the simple improved Tokuhashi scoring system. This might be attributed to the thorough preoperative review of patient data by the MDT, detailed surgical planning, collaborative efforts among different departments during surgery, prompt completion of the procedure, a reduced risk of major intraoperative bleeding, appropriate pain management postoperatively, proactive prevention of wound infection, and early rehabilitation training. However, there were no statistically significant differences between the two groups in terms of cerebrospinal fluid leakage (2 patients, 3.85%) or neurological impairment (1 patient, 1.92%). This could be because the general clinical data of the patients included in both groups were comparable, and the surgeries were performed by experienced spine surgeons. Deep vein thrombosis (7.69%) was the most common complication and was likely associated with prolonged bed rest after surgery. The average length of hospital stay postoperatively was shorter (7.94 ± 0.28 days), and the long-term follow-up showed significant improvements in VAS scores, Karnofsky Performance Status (KPS) percentage scores, Frankel grading for neurological function, Eastern Cooperative Oncology Group (ECOG) physical condition grading, and SAS anxiety self-rating scale scores compared to those of patients in the group involving use of the simple improved Tokuhashi scoring system (P < 0.05). This can be attributed to the collaborative efforts of the MDT, which focuses on patient-centred care, not only treating patients’ physical conditions but also providing psychological support to facilitate rapid recovery and enhance their quality of life. As Gasbarrini et al. [19] suggested in their study on the treatment evaluation process of 182 patients with spinal metastases, a multidisciplinary approach enables the implementation of more effective treatment plans and improves the quality of life for these patients.

Therefore, surgical treatment of spinal metastases using a combined improved Tokuhashi scoring decision system by a multidisciplinary team (MDT) has several advantages. (1) The expertise of professionals from different departments is fully utilized to develop the optimal treatment plan. The MDT approach involves collaboration between orthopaedics, oncology, radiation therapy, pain management, nuclear medicine, endocrinology, pathology, psychosomatic medicine, and other disciplines. Through multidisciplinary evaluation considering the patient’s physical condition, organ function, examination results, and relevant scores, a standardized and personalized treatment plan was formulated using the NOMS decision system and the improved Tokuhashi scoring system. This allows for comprehensive and individualized treatment at the appropriate time, maximizing patient benefits [8]. (2) It significantly improves patients’ quality of life, reduces hospitalization time, decreases intraoperative and postoperative complications, and lowers patient mortality rates. (3) It emphasizes the humanistic aspect of healthcare, enhancing patients’ confidence in overcoming their illness. The multidisciplinary approach focuses on both the physical and mental aspects of the disease, providing timely attention to patients’ psychological changes through counselling and treatment. This enhances patients’ confidence in fighting the disease, promotes their recovery, and fosters a positive doctor‒patient relationship [20]. (4) The improved Tokuhashi scoring system, based on a classification prognosis model, was combined with the principle-based NOMS scoring decision system to create a rational and standardized surgical decision system. The need for surgery for spinal metastases and the choice of surgical approach have not yet been standardized [21, 22]. Using the multidisciplinary team and the NOMS decision system in combination with the improved Tokuhashi scoring system, individualized, precise, and rational treatment can be provided for patients.

The limitations of this study mainly include the following: (1) The surgical treatment group with a pure NOMS was not included in the study. We will include them in the later stage of the research. (2) The sample size of this study was relatively small, and we will continue to expand the sample size in the future. (3) The follow-up time was insufficient, and we extended the follow-up time in the later stage. (4) The Kaplan‒Meier method was used to calculate the survival rate, but Cox regression analysis of related factors between the two groups was not conducted. We will include them in the analysis in the later stage.

## Conclusion

In summary, the multidisciplinary team using the NOMS and Revised Tokuhashi scoring systems, achieved better clinical efficacy in treating spinal metastasis than did the Revised Tokuhashi system alone, offering more individualized, precise, and rational treatment, improving patient quality of life, shortening hospital stays, and reducing complications. This approach is worthy of clinical promotion.

## Data Availability

All data generated or analyzed during this study are included in this published article.

## Abbreviations

MDT: Multidisciplinary Team
VAS: Visual Analogue Scale
ECOG: Eastern Cooperative Oncology Group
SAS: Self-rating Anxiety Scale
KPS: Karnofsky Performance Status
SINS: Spinal Instability Neoplastic Score
cEBRT: conventional External Beam Radiation Therapy
SRS: Stereotactic Radiosurgery
ESCC: Epidural Spinal Cord Compression
